# Principal components analysis based methodology to identify differentially expressed genes in time-course microarray data

**DOI:** 10.1186/1471-2105-9-267

**Published:** 2008-06-06

**Authors:** Sudhakar Jonnalagadda, Rajagopalan Srinivasan

**Affiliations:** 1 Department of Chemical and Biomolecular Engineering, National University of Singapore, 10 Kent Ridge Crescent, Singapore

## Abstract

**Background:**

Time-course microarray experiments are being increasingly used to characterize dynamic biological processes. In these experiments, the goal is to identify genes differentially expressed in time-course data, measured between different biological conditions. These differentially expressed genes can reveal the changes in biological process due to the change in condition which is essential to understand differences in dynamics.

**Results:**

In this paper, we propose a novel method for finding differentially expressed genes in time-course data and across biological conditions (say *C*_1 _and *C*_2_). We model the expression at *C*_1 _using Principal Component Analysis and represent the expression profile of each gene as a linear combination of the dominant Principal Components (PCs). Then the expression data from *C*_2 _is projected on the developed PCA model and scores are extracted. The difference between the scores is evaluated using a hypothesis test to quantify the significance of differential expression. We evaluate the proposed method to understand differences in two case studies (1) the heat shock response of wild-type and HSF1 knockout mice, and (2) cell-cycle between wild-type and Fkh1/Fkh2 knockout Yeast strains.

**Conclusion:**

In both cases, the proposed method identified biologically significant genes.

## Background

Microarray expression profiling is often carried out to identify genes whose expression change across biological conditions [1]. Two types of expression profiling can be differentiated, static and time-course. In the static type, snapshots of gene expression levels are measured in two different cell populations, such as normal and diseased [2]. Genes that are differentially expressed in the diseased cells, compared to normal cell population, disclose pathways related to the disease and also serve as signature of the disease. However, measuring expression levels irrespective of time does not provide information about the dynamic interactions that characterize the cellular processes [3]. This necessitates time-course experiments where gene expression levels are measured at different time-points and across biological conditions such as wild-type and gene-knockout strains [4] or normal and stimulated cells [5].

Several methods have been proposed in literature to identify differentially expressed genes in static experiments. The simplest technique is the calculation of fold change of gene expression between the conditions. Genes with fold change above a user-defined threshold such as 2-fold change may be considered as differently expressed. For cases where replicates of microarray experiments are available, methods based on standard t-test, modified t-test and non-parametric tests have been proposed (see reviews [6] and [7]). These methods are not directly applicable for time-course experiments where differential expression has to be calculated globally in the temporal space and not just between corresponding time points [8].

Recently, several methods have been proposed to identify differentially expressed genes in time-course data. Bar-Joseph et al. [9] proposed a method that represents expression profiles as continuous curves and then uses a global difference between the curves to identify differentially expressed genes. In their approach, clustering of genes is used as a preprocessing step; although simple, this makes the method computationally expensive for large datasets. Storey et al. [8] proposed a method that measures the improvement in goodness-of-fit when a single curve is used to fit the data from both conditions compared to fitting a separate curves for each condition. If the improvement in goodness-of-fit is significant then that particular gene is considered as differentially expressed. Their approach treats all genes as equal irrespective of their expressions levels in the experiments. This leads to the spurious identification of genes with low expression in both conditions as differentially expressed genes (see results). Conesa et al. [10] proposed a regression-based approach that models the expression profile of each gene with time as regressor and tests the hypothesis on the equality of regression coefficients. A similar method is proposed by Vinciotti et al. [11] where the expression profiles are fitted using cubic polynomials and tested for similarity of coefficients. Modeling individual genes is generally not recommended due to noise in the microarray data [12]. Cheng et al. [13] proposed an approach that represents the time-course data from both conditions as two different gene relationship networks where each node is a gene and each edge links the two similarly expressed genes. Differentially expressed genes are identified by comparing the neighborhood of each gene *i *in both networks. The neighborhood of gene *i *consists of genes that have similar expressions. Genes with dramatic change in neighborhood are deemed as differentially expressed. Since the actual expression of a gene is not directly compared in both conditions, genes similarly expressed in both conditions can be declared as differentially expressed if their neighbors change. Reverter et al. [14] proposed a method that identifies genes that are simultaneously differentially expressed and differentially connected. However, they quantify the difference in expression of a gene as the sum of differences in individual time-points. This may not capture systematic variations. Methods based on ANOVA [15] and ANCOVA [16] models have also been proposed specifically for replicated time-course data.

Each one of the available methods for identifying differentially expressed genes in time-course data have particular drawbacks. They also do not consider natural dependencies among different time-points. The noise in the data is also ignored. In this paper, we propose a statistical method for identifying differentially expressed genes in time-course data. The proposed method uses Principal Components Analysis (PCA) to consider the correlation among different time-points and reveal fundamental patterns in the data. The scores of genes on these fundamental patterns are used to identify the differentially expressed genes. Noise is discounted by considering only the most significant PCs (patterns) in the analysis.

Let time-course gene expression be measured at two different biological conditions, *C*_1 _and *C*_2_. The proposed method relies on Principal Components Analysis (PCA) to model the expression data from *C*_1_. Noise is removed from the model by using only the dominant components. When the expression data from *C*_2 _is projected on this PCA model, differences in the gene expression program can be identified. Genes whose expressions do not change between the two conditions will have similar scores, while scores will be different for differentially expressed genes. A statistical test is used to find the significance of the difference in scores and reliably identify differentially expressed genes and their p-value (see Methods section for details).

There are several advantages of using PCA for finding differentially expressed genes: (1) The score of a gene on a PC is the correlation between the gene and the PC. Comparing the scores is equivalent to comparing the similarity of temporal expression profiles. So the proposed approach uses the systematic differences in expression to identify differentially expressed genes, (2) Since only the dominant PCs are used for analysis, the effect of noise in the data is alleviated. This leads to meaningful comparison of expression profiles across conditions and identifies significant differentially expressed genes. (3) PCs are the fundamental patterns in the data. They can be interpreted and hence provides more information about the differences in expression of genes [17-19].

We evaluate the proposed method using two case studies. The first case study involves genome-wide study of differences in the heat-shock response of wild-type mouse and mouse lacking heat-shock transcription factor 1 (HSF1). The second case study concerns the Yeast cell-cycle response between the wild-type and a mutant lacking forehead proteins (Fkh1 and Fkh2). We compare the results from these studies with results from other recent approaches.

## Results

We test our approach using two publicly available datasets. The first time-course dataset is from heat-shock response of wild-type and HSF1 mutant mice. The second dataset is from Yeast cell-cycle study in a wild-type and Fkh1 and Fkh2 double mutant strain.

### Case Study 1: mouse time-course dataset

Heat-shock transcription factor 1 (HSF1) is the primary regulator for many heat-shock proteins in mammalian cells. To characterize its role, Trinklein et al. [20] measured the transcription levels and also assayed the binding of HSF1 on human promoters. From this study, Trinklein et al. [20] hypothesized that the induction of several heat response genes is independent of HSF1. To test the hypothesis, Trinklein et al. [20] measured the expression levels of 9468 mouse genes using cDNA microarrays. Expression levels of genes are measured at 0, 0.5, 1, 2, 3, 4, 6, and 8 h after the heat-shock in both wild-type and mouse lacking HSF1. Trinklein et al. [20] analyzed the transcriptional response of different gene groups: (A) mouse genes homologues of human genes that are bound by HSF1 and induced, (B) homologues that were bound by HSF1 but not induced, (C) homologues that were induced but not bound by HSF1, (D) genes induced by heat in wild-type but not in mutant, (E) genes induced in mutant mouse, (F) genes induced similarly in both wild-type and mutant. Ideally, genes belonging to groups A, D and E should be identified as differentially expressed between wild-type and HSF1 mutant mouse and genes belonging to groups C and F as similarly expressed.

#### Modeling the wild-type time-course data

**Figure 1:**
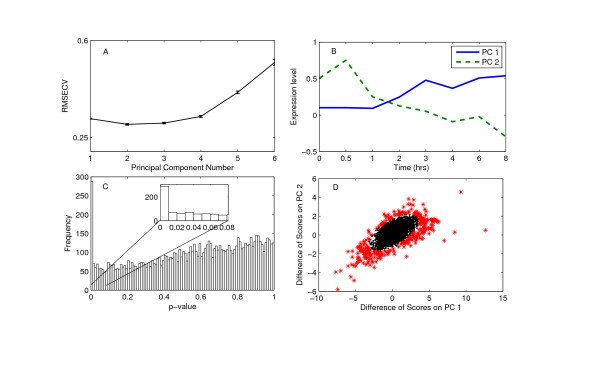
**Results for mouse dataset**. (A) Cross-validation results for the wild-type mouse time-course data. The RMSECV has the minimum value at number of PCs 2. So two PCs are used to model this dataset. (B) The PCs extracted in the wild-type mouse dataset. First PC shows the pattern related to activation of genes. The second PC has increased expression in the first time-point and then decreases. It corresponds to the dynamic changes in genes expression due to heat-shock. (C) The distribution of p-value of the genes in mouse dataset. There are 288 genes in the p-value range 0–0.01. After that the distribution if more or less uniform. The p-value threshold selected for this dataset is 0.01. (D) Difference of scores of mouse genes on first two PCs. The differentially expressed genes identified by the proposed method are marked '*'.

We modeled the time-course expression data from the wild-type mouse using PCA. The number of PCs, *k*, to be retained in the model was found using cross-validation. The root-mean square error of cross-validation (RMSECV) takes the minimum value at *k *= 2 (Figure 1A). The first two PCs capture 42.12% and 24.75% of the total variance, respectively. The third PC captures only 9% of the variance and the remaining PCs smaller amounts. The expression profiles of PCs are shown in Additional file 1. The first two PCs model systematic changes in expression where as rest appear to have random expressions depicting noise. This provides additional evidence that the first two PCs capture most of the variance and the rest of the PCs essentially contain the noise in the data. So, selection of two PCs is justified for this dataset. In order to validate the PCA model, we analyzed the expression profiles of these two PCs shown in Figure 1B. In wild-type mouse, the heat-shock activates several heat inducible genes. The first PC shows an upward trend indicating the activation of the genes due to heat-shock. Genes whose scores are positive on this PC show similar trend in their expression. Some of these genes include heat inducible genes hsp60, hsp70, hsp86, etc. The second PC shows an upward trend at 0.5 hrs after the heat-shock and shows a downward trend afterward. This PC represents the dynamic changes in the expression of genes over time.

#### Identifying differentially expressed genes

The time-course data from the mouse lacking HSF1 is projected on the developed PCA model and the scores of these genes on the two PCs are extracted. The differences in their scores are used to calculate the p-values for the genes (see Methods section for details). The histogram of the p-values for all the genes is shown in Figure 1C. There are 288 genes in the p-value range 0–0.01. The frequency drops to 70 in the range 0.01–0.02 (see the inset in Figure 1C) and the p-values for the rest of the genes are distributed more or less uniformly. So, we selected a p-value threshold of 0.01 for this dataset.

The proposed method identifies 288 genes as differentially expressed at this p-value threshold. The differences in the scores on two PCs are shown in Figure 1D. The differentially expressed genes (marked as '*') are far away from the majority of the genes. This confirms that the proposed hypothesis test identifies the genes with large difference in scores. Since the HSF1 gene is knocked-out in the experiment, we expect that the targets of HSF1 gene will be differentially expressed in the mutant mouse. On the other hand, genes related to metabolism and signaling processes are expected to be similarly expressed in the wild-type and mutant mice. The differentially expressed genes identified by the proposed method include genes previously reported as the targets of the HSF1 such as hsp60, hsp70, hspa8 [21]. In contrast, several metabolic and signal transduction genes including methylene tetrahydrofolate dehydrogenase, carbon catabolite repressor, Protein kinase C alpha binding protein, and MAD homologue 7 are not identified as differentially expressed. The p-values for these genes are between 0.018–0.9989. This clearly shows that the proposed method is able to identify differentially expressed genes with biological implications.

Our method identifies four (out of 9), group A mouse genes homologues of human genes that are both bound by HSF1 and induced in wild-type mouse. These are Hsp105, Dnajb1, hsp84-1, and Cacybp and the corresponding p-values are 1.0 × 10^-15^, 7.014 × 10^-8^, 3.0614 × 10^-4^, and 4.7355 × 10^-4^. On the other hand, 13 (out of 15) group C mouse genes homologue to human genes that are induced in wild-type but not bound by HSF1 are not identified as differentially expressed genes. The p-values for these genes are in the range of 0.035–0.927. These results support the hypothesis that HSF1 does not regulate all the heat induced genes.

#### Comparison of results with previous study

**Figure 2:**
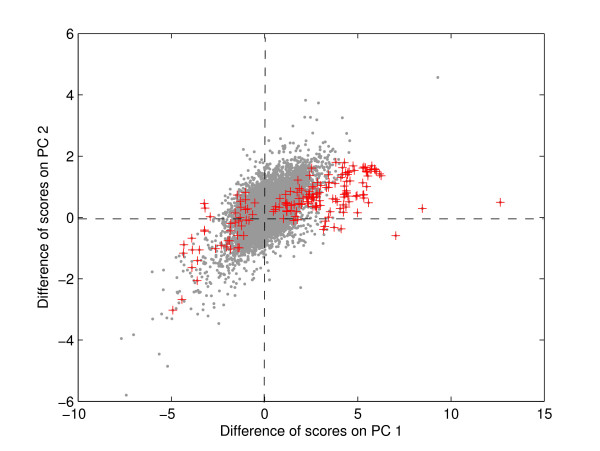
**Difference of scores of mouse genes on first two PCs**. The differentially expressed genes identified by Trinklein et al. [20] are marked '+'. These genes span mainly in the direction of the first PC. The first PC represents the pattern related to activation of genes. Genes on the positive side of the plane are up-regulated in wild-type and down-regulated in mutant mouse. Genes on the negative side of the plane are down-regulated in wild-type and up-regulated in mutant mouse. This indicates that Trinklein et al. [20] identified only the genes that are completely up- or down-regulated.

Trinklein et al. [20] reported 167 genes differentially expressed in the experiment (groups D and E). Our approach identified 78 of the genes out of these 167. Most of the remaining genes identified by Trinklein et al. [20] have <2-fold change at all the time-points in both wild-type and the mutant mouse. Trinklein et al. [20] used the heatmaps of the clusters to identify differentially expressed genes. In heatmaps, small positive and small negative values are showed in different colors and can hence lead to mis-identification as differentially expressed. The proposed approach also identified 210 novel genes as differentially expressed. We clustered these genes using hierarchical clustering [see Additional file 2]. The figure shows the novel genes are differentially expressed between the wild-type and mutant mouse. Trinklein et al. [20] identified the genes that were completely up- or down-regulated between the wild-type and mutant mice. This can be seen in Figure 2 where the genes identified by Trinklein et al. [20] span only in the direction of first PC that represents activation of genes after heat-shock. The proposed approach identifies all the genes with differential expression between the two mice.

### Case Study 2: Yeast cell-cycle dataset

For the second case study, we use the Yeast cell-cycle dataset where the expression levels of genes are measured over two cell-cycles in a wild type and Fkh1, Fkh2 double mutant strain. Spellman et al. [22] monitored the expression levels of almost all genes during two cell-cycles. Eighteen samples were taken following the *a *factor release with a sample period of 7 mins. They identified 800 cell-cycle regulated genes using periodic algorithms. Zhu et al. [4] monitored the expression levels of Yeast genes in a mutant strain that lacks two forkhead transcription factors Fkh1 and Fkh2. They measured expression levels at 13 time-points, the first twelve at 15 min intervals from time 0 till 165 mins, and the last at 210 mins. Out of the 800 cell-cycle genes reported by Spellman et al. [22] in the Wild-Type (WT) strain, expression data is available for 746 genes in the Knock-Out (KO) experiment. So we use the expression data for these 746 genes from both strains to evaluate the proposed method.

Since the number of samples and the time of samples are different in WT and KO experiments, we use dynamic time warping [23] to align the expression profiles by warping their time scales. Particularly, we use asymmetric time warping algorithm to map the time axis of the KO genes signals to the WT ones. The expression profiles of both the WT and KO genes are fitted to cubic splines and resampled at each minute. These supersets are aligned using asymmetric DTW. After alignment, the resampled expression values for the KO are obtained at the time points corresponding to the original WT samples (0 to 119 mins with a period of 7 mins). The aligned datasets for both the WT and KO strains thus contain expression of 746 genes at 18 time points.

#### Modeling the wild-type time-course data

**Figure 3:**
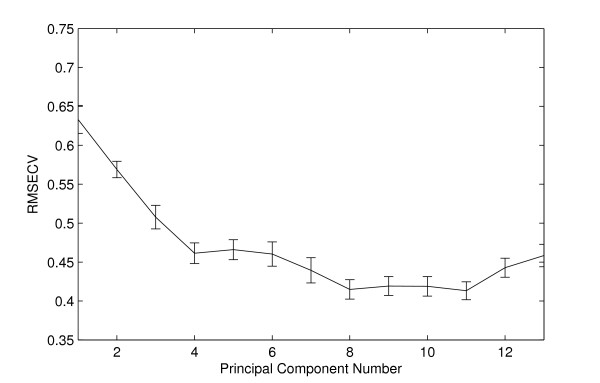
**Cross-validation results for wild-type yeast cell-cycle dataset**. The RMSECV takes local minima at number of PCs 4, 8 and 11. The first 4 Principal components (PCs) captured almost 80% of the variance in the data and are used to model this dataset.

**Figure 4:**
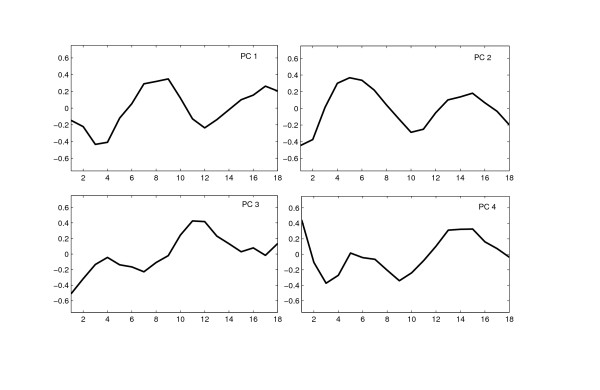
**Principal Components extracted from the wild-type Yeast cell-cycle dataset**. The four PCs extracted from the wild-type Yeast cell-cycle dataset have distinct patterns and map to different phases of the cell-cycle.

We modeled the expression time-course data from the wild-type Yeast strain using PCA. The RMSECV has local minima at *k *= 4, 8 and 11 (Figure 3). The expression profiles of all PCs are shown in Additional file 3. The first four PCs have systematic changes in expression. The first 4 PCs capture approximately 80% of the variance in the data. Considering the noise levels in microarray data, we use only 4 PCs. The expression profiles of the four PCs are shown in Figure 4. These PCs correspond to different fundamental patterns in the WT cell-cycle data. Genes from different phases are found to be highly correlated with these patterns. For example, genes with high scores on PC 1 such as Clb2, Clb1, Ace2 and Cdc5 are mainly from G2 and M phases. Similarly, genes from G1 and S phases have higher scores on PC 2, the PC 3 maps to the M/G1 and G2 phases. PC 4 contributes to genes from different phases.

#### Identifying differentially expressed genes

When the resampled KO (*C*_2_) gene-expressions were projected to the PCA model, the proposed method identified 72 genes as differentially expressed at the p-value threshold of 0.05. We identified several genes expressed at high levels in WT strain but showing little or no expression in KO strain. For example, 40 genes had 2-fold change in at least one time-point in the WT strain that lost their expression in the KO strain and showed less than 2-fold change in all time-points. The proposed method also identified 4 genes that have less than 2-fold change in WT strain but having 2-fold change at one time-point (2 genes) and 2 time-points (2 genes) in the KO strain. We identified one gene that has less than 2-fold change in both WT and KO strain as differentially expressed. All the remaining genes showed high expression levels in both the WT and KO strains but differed in their expression profiles.

**Figure 5:**
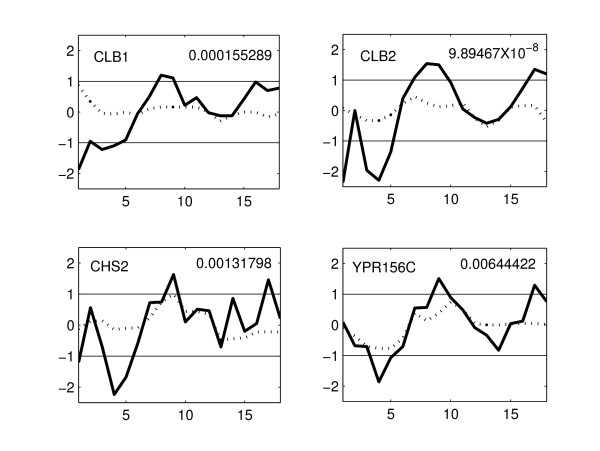
**Expression profiles of four genes identified by the proposed method in the CLB2 cluster**. The solid line represents the expression of gene in the WT and the dotted line represents the expression of gene in the KO strain. Gene names and the p-values are shown for all genes. The WT genes show an oscillatory behavior while the expression in KO is significantly changed.

**Figure 6:**
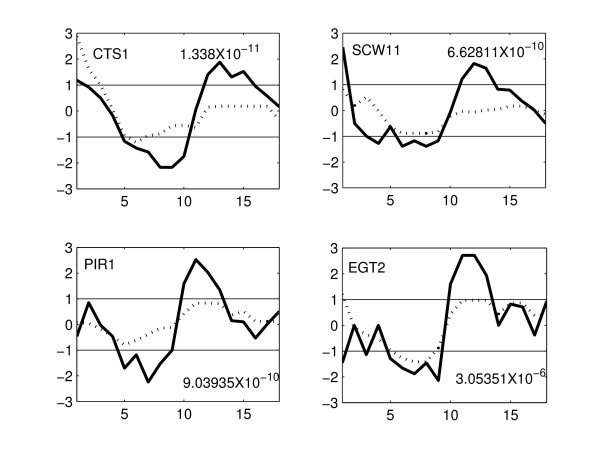
**Expression profiles of four genes identified by the proposed method in SIC1 cluster**. The solid line represents the expression of gene in the WT and the dotted line represents the expression of gene in the KO strain. Gene names and the p-values are shown for all genes. There is considerable change in the expression of SIC1 genes between WT and KO strain.

Zhu et al. [4] analyzed the heatmaps of clusters of co-expressed cell-cycle genes and reported that genes from CLB2 and SIC1 clusters are differentially expressed in the mutant strain. The proposed method identifies several genes from CLB2 and SIC1 clusters. We identified 11 genes (out of 31) from CLB2 cluster. The expression profiles of four of these genes in WT and KO are shown in Figure 5. These genes show a significant difference in their expression between the WT and KO strains – oscillatory behavior (with > 2-fold change) in the WT strain and almost no expression in KO strain. Some of the remaining genes in this cluster have flat expression profiles in the KO as well as in WT [see Additional file 4]. The genes identified by the proposed method are the most significantly differentially expressed genes in CLB2 cluster. In the SIC1 cluster, we identified 16 (out of 26) genes. The expression profiles of some of these genes in WT and KO are shown in Figure 6. From this figure, it is clear that the genes identified are differentially expressed. The remaining 10 genes showed a little expression in both the WT and KO [see Additional file 5]. The benefit of the proposed method is the quantitative comparison of the expression profiles which enables the identification of significantly differentially expressed genes and eliminates subjective errors.

We validate the results at different levels. First, we compare the genes identified by the proposed method with results from other approaches for identifying differentially expressed genes. The novel genes identified by our method are evaluated using the Genome-wide location data from Simon et al. [24] who studied genome-wide transcription factor (TF)-DNA interactions for nine cell-cycle TFs including Fkh1, Fkh2, Ace2 and Swi5. Finally, differential expression of genes is also confirmed by directly comparing the actual expression profiles.

#### Comparison with results from other methods

We compare our results with the results from the different approaches proposed for identifying differentially expressed genes in time-course microarray datasets. Bar-Joseph et al. [9] used the same datasets and reported 56 genes as differentially expressed. There is a significant overlap between the genes identified by our method and those reported by Bar-Joseph et al. [9]. Our method identifies 44 of these 56. Changing the p-value threshold to 0.1 includes 5 more genes. We found all the genes identified by Bar-Joseph et al. [9] in CLB2 cluster. Additionally, our list includes Cdc5 and YPR156C from that cluster. Cdc5 is a pole-like kinase, possibly a substrate of Cdc28, which is found to be bound by Ndd1. Even though Ndd1 is not directly affected in this experiment, its binding is mediated by Fkh2 in G2/M [25]. The second gene YPR156C is involved in polyamine transport. There are no regulators found to be bound to this gene in TF-DNA interaction data. However, its expression is different between WT and KO. Similarly, most of the genes reported by Bar-Joseph et al. [9] from the SIC1 cluster have been identified by our method.

We used the EDGE software by Storey et al. [8] to identify differentially expressed genes. Using natural cubic splines with basis of 4, their method identifies 73 genes as differentially expressed at the p-value threshold of 0.001. Only 30 (out of these 73) genes match the genes identified by our method, and only 22 genes with those identified by Bar-Joseph et al. [9]. Overall, 21 genes are identified by all the three methods, while 42 are novel genes identified only by the Storey et al. [8] approach. Most of these novel genes show very little expression in both the WT and KO strain [see Additional file 6]. Only 7 of the 42 novel genes are found to be bound by one or more of Fkh1, Fkh2, Ace2 and Swi5. The normalization procedure they use equally weighs highly expressed genes and genes with little expression. This is the probable reason for the misidentification of genes with little expression as being differentially expressed.

Recently, Cheng et al. [13] used the cell-cycle dataset to evaluate their approach and identified 100 genes as differentially expressed, among which 41 genes are present in out dataset (we used 746 cell-cycle regulated genes). We identified 19 out of these 41 genes as differentially expressed. Additional 6 genes will be identified as differentially expressed if the p-value threshold is increased to 0.1. The expression profiles of the remaining 22 genes are show in Additional file 7. Several genes showed similar expression in both wild-type and the mutant strain. The approach proposed by Cheng et al. [13] considers the change in neighborhood of a gene in two conditions. Since the actual expression profile of genes is not compared in different conditions, genes with similar expression profiles could also be detected as differentially expressed if their neighborhood genes are differentially expressed.

#### Validation of Novel genes

Using our method, we identified 28 novel genes that have previously not been identified. We find the TFs for the novel genes using Genome-wide location data from Simon et al. [24] with a strict p-value threshold of 0.005 for TF-DNA binding (Table 1). The novel genes we identified are from all cell-cycle phases. It is known that cell-cycle is carried out by serial regulation of transcription factors [24]. So it is expected that a change in the cell-cycle will affect the different phases. 13 genes (out of 28) are found to be bound by one or more of Fkh1, Fkh2, Ace2, and Swi5. Fhk2 is the predominant binding partner for Mcm1 and it also mediates the binding of Ndd1 [25]. So genes regulated by Mcm1 or Ndd1 would possibly change their expression in the mutant strain. The remaining genes are found to be bound by one or more of Swi4, Swi6, and Mbp1. Both Swi6 and Mbp1 have very little expression in WT and they were not identified as cell-cycle regulated genes by Spellman et al. [22]. So, the data we used includes only Swi4. The p-value for Swi4 is 0.06 which is very close to the threshold we used. It also shows a difference in expression between WT and KO. This differential expression of Swi4 is probably the reason for the differential expression of genes bound by it.

**Table 1:** Validation of novel genes

Gene	Phase	p-value	Transcription Factors
PCL9	M/G1	0.0495	Swi5
CHS1	M/G1	0.0098	Swi5
YDL117W	M/G1	0.0110	
YBR296C	M/G1	0.0104	
SST2	M/G1	0.0224	
AGA1	M/G1	0.0048	Mcm1, Mbp1, Swi4, Swi6
TSL1	G1	0.0274	Fkh1, Fkh2, Ndd1, Ace2, Swi5, Mbp1, Swi4, Swi6
CLB6	G1	0.0027	Fkh2, Mbp1, Swi4, Swi6
SVS1	G1	0.0001	Fkh1, Fkh2, Swi4, Swi6
POL30	G1	0.0268	
MCD4	G1	0.0257	
YOX1	G1	0.0085	Fkh2, Mbp1, Swi4, Swi6
CLN2	G1	0.0088	Swi6
YMR305C	G1	0.0187	Mcm1, Mbp1, Swi4, Swi6
HHT1	S	0.0403	Fkh2
HHO1	S	0.0162	Swi4, Swi6
YIL129C	G2	0.0021	Swi5
YMR215W	G2	0.0025	Fkh1, Fkh2, Mbp1, Swi6
CIK1	G2	0.0126	Fkh1, Fkh2
CDC5	M	0.0033	Ndd1
YPR156C	M	0.0064	
YPR157W	M	0.0219	
NCE2	M	0.0203	Fkh2, Ndd1, Swi4
FET3	M	0.0042	
YOR383C	M	0.0371	
YDL039C	M	0.0089	
CLN3	M	0.0108	Mcm1, Ace2, Swi5, Swi4, Swi6
MFA2	M	0.0216	Fkh1, Ndd1, Mcm1, Swi5

Novel differentially expressed genes identified by the proposed method. Genes are grouped based on the phase of the cell-cycle where they show peak expression.

#### Understanding cell-cycle using novel genes

**Figure 7:**
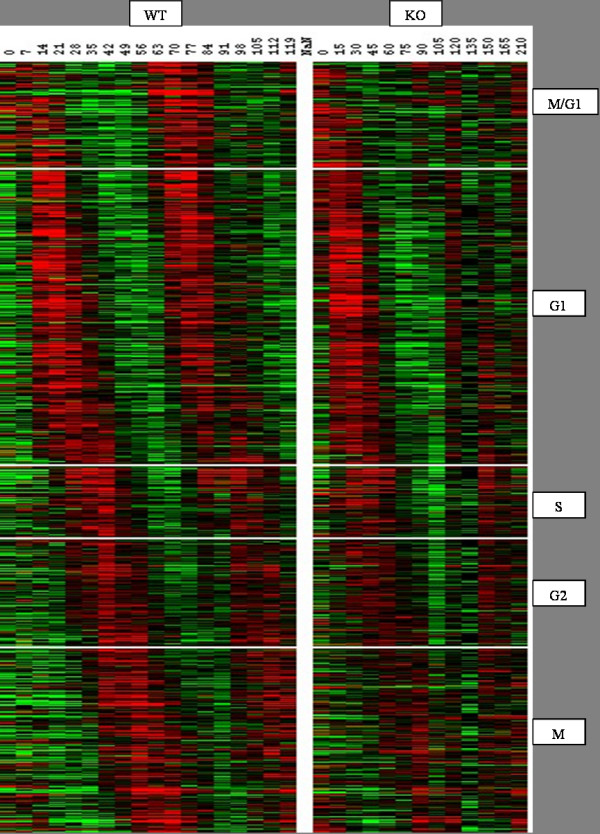
**Heatmap of cell-cycle expression data from WT and KO strains**. Most of the genes from M/G1 and M phases are differentially expressed in the KO strain compared to the WT strain. Genes from G1 phase retained their expression during first cell-cycle but are differentially expressed in second cell-cycle. Most of the genes from G2 and S phase show little or no change from their WT expression.

To understand how the cell-cycle is affected by the deletion of the two forkhead proteins Fkh1 and Fkh2, we constructed a heatmap of the cell-cycle regulated genes using the Treeview software [26](Figure 7). As expected, genes having peak expression in M (CLB2 genes) and M/G1 (SIC1 genes) phases of cell-cycle have lost their expression in the KO strain. Several G1 genes also showed a significant difference in their expression. One interesting aspect we observed in the heatmap is that, in the KO strain, most of the genes from G1 phase retained their expression in the first cell-cycle but not in the second cycle. However, the phenotype indicates that cells entered into second cell-cycle: mother and daughter cells budding synchronously [4]. The novel genes we identified as differentially expressed partially explain this phenomenon.

**Figure 8:**
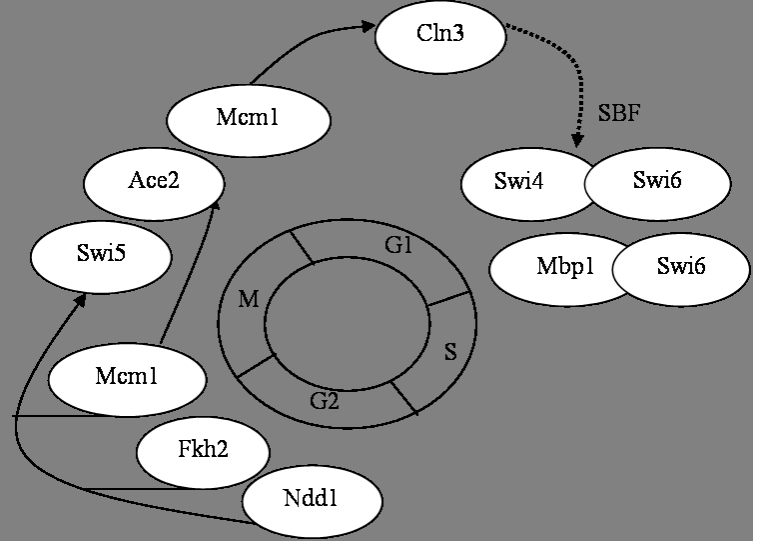
**Simple model of cell-cycle-regulation of Yeast**. Transcription factors (TF) that regulate genes from different phases of cell-cycle are represented as ovals and placed near the corresponding phases. Solid lines represent the regulatory interaction and the dotted line represents the post transcriptional actions.

To understand the cell-cycle regulation in Yeast, consider Figure 8, a simplified form of Simon et al. [24] cell-cycle model. Two transcription factor complexes SBF (complex of Swi4 and Swi6) and MBF (complex of Mbp1 and Swi6) are major regulators of G1 phase genes. SBF requires Cln3-Cdc28 to change to active state by post-transcriptional action [27].

**Figure 9:**
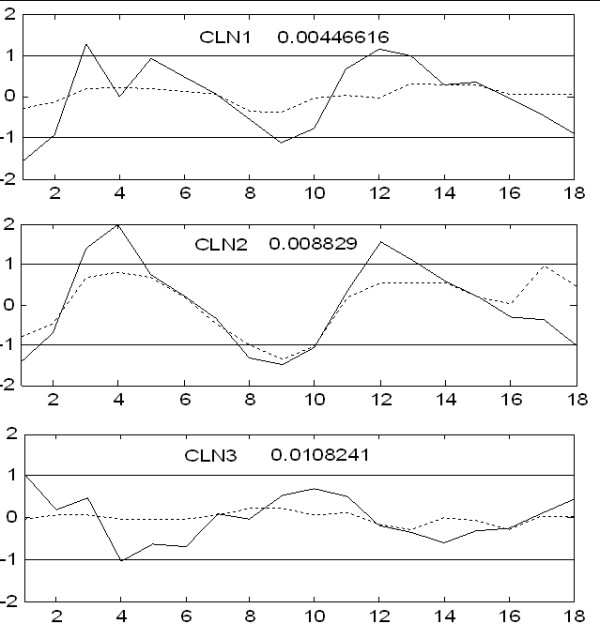
**Expression profile of three CLN genes in WT and KO strain**. Cln1 loses its oscillatory behavior and is almost flat in KO strain. Cln2 retains its oscillation but at a diminished magnitude. Cln3 is not expressed in the KO strain. Only Cln1 is reported previously as differentially expressed. We identified the remaining two CLN genes.

In contrast to the other approaches which identify only Cln1, we identified all three CLN genes (Cln1, Cln2 and Cln3) as differentially expressed. The expression profiles of these three genes are shown in Figure 9. In the WT strain, all three show oscillatory behavior. Cln1 loses its oscillatory behavior in the KO strain and its expression is very low. Cln2 retains its oscillatory behavior but at a lower magnitude. Cln3 is not expressed in the KO strain. Cln3 is found to be bound by Mcm1, Ace2, Swi5, Swi4 and Swi6 (Table 1). So we hypothesize that for the KO strain, expression of Cln3 is affected, because of which SBF is in an inactive state. Consequently, the expression of G1 phase genes during the second cell-cycle is altered. It has been reported that the other two CLN genes (Cln1 and Cln2) are regulated by SBF [28]. The significant decrease in their expressions in the KO strain also lends evidence to the hypothesis that Cln3 affected SBF which in turn affected several G1 phase genes in the second cell-cycle (Figure 7). Further evidence is that CLB6, which is bound by SBF (Table 1), is also identified as differentially expressed.

## Discussion

In both the case studies, the Wild-Type (WT) dataset was modeled using PCA and the Knock-Out (KO) data was projected on the model. When the KO data is used for model development and WT data projected on the model to identify differentially expression genes, the results are almost the same. For the Yeast cell-cycle Case study 5 PCs are needed to model the Knock-out data [see Additional file 8]. With this model, 89 genes were detected as differentially expressed at a p-value threshold of 0.05. There is a significant overlap between the two sets. Out of 72 genes from the WT model, 69 were also identified by the KO model. The median rank of these 72 genes is 37.5 which is very close to median rank of 36.5 if all these 72 are in the top in the list. This indicates that the almost same genes are identified as differentially expressed in both scenarios and the proposed method is robust.

The proposed method uses a hypothesis test to find the significance of the differential expression of a gene between two biological conditions. This test assumes that difference of scores between WT and KO follows a multivariate normal distribution. The scores are the weighted linear combination of original expressions (Eq 4). As per the central limit theorem, linear combinations of variables would follow a normal distribution even if the individual variables do not. If scores are normally distributed, so would their difference. We tested the normality of the difference of scores on each PC using quantile-quantile plots for both the mouse dataset [see Additional file 9] and Yeast cell-cycle dataset [see Additional file 10]. The coefficient of determination between the observed values and the expected values ranges from 0.92 to 0.97. We also tested the multivariate normality using beta probability plot of Small [29]. The coefficient of determination, using all genes, is 0.65 for mouse dataset which further increases to 0.95 after removal of only 1% of outlier genes [see Additional file 11]. Similarly, for Yeast cell-cycle dataset the coefficient of determination is 0.81 when all genes are used and 0.96 after removal of 5% outlier genes [see Additional file 12]. Hence, the assumption of the normality is reasonable.

The proposed method uses Mahalanobis distance as the distance metric to find differentially expressed genes. Mahalanobis distance is the most widely used distance metric with PCA analysis. It weighs different directions(PCs) differently and the weights are inversely proportional to the variance in those directions. So, differences in expression in directions with large variance (inherently higher noise) are given less credit when identifying differentially expressed genes. However, this assumes that all co-expressed genes are similar in magnitude. This assumption may not be valid particularly for Transcription Factors (TFs) since their expression levels are often much lower than other genes. For example, the p-value for TF ACE2 in Yeast cell-cycle case study is 0.1794. The expression levels of ACE2 are lower compared to its co-expressed genes such as ALK1, CLB1, and IQG1. The later genes are identified as differentially expressed by the proposed method. This is common shortcoming for methods based on quantitative analysis. It is better to visualize the actual expression profiles for TFs rather than selecting a strict p-value threshold or suitable normalizing techniques can be used in processing step.

The proposed method currently does not include replicates information. Replicates improve the reliability of identifying differentially expressed genes. It is possible to extend the method using ideas from Multiway Principal Component Analysis (MPCA) to explicitly include replicates.

Finally, the proposed method is useful especially for large datasets since it relies on PCA which is computationally efficient even for large number of genes. In large datasets, most of the genes are generally unchanged between different biological conditions. Consequently, the differential expression may not be reflected in all dominant PCs as the PCs are not driven by differential expression between different conditions. Yet, the proposed method identifies differentially expressed genes correctly. To illustrate this, we used the complete dataset containing all cell-cycle- and non-cell-cycle-regulated genes. The datasets contain measurements for 5696 genes at 18 time points. Considering the large number of genes, a more stringent p-value threshold of 0.001 is used instead of 0.05 that was used for the cell-cycle genes. We identified 151 genes as differentially expressed which contained 68 (out of 72) genes identified in the cell-cycle data alone.

## Conclusion

In this paper, we proposed a method for identifying differentially expressed genes in time-course data. We evaluated the proposed method using two gene expression datasets and compared the results with previously published results. The proposed method models the expression data from one condition using PCA and projects the expression data from another condition on the developed PCA model. The scores of genes are used to identify differentially expressed genes. Since scores represent the linear relation between the expression profile of genes and the PC, comparison of scores measures the systematic variation in the gene expressions. In contrast to previously published methods that treat all the genes equally irrespective of actual expression levels [8], directly compare the expression profiles [9], or not use the expression levels [13], our approach uses PCA where different PCs contribute differently to the gene expression profiles and provide comparison at multiple levels. This is important because, for some genes a small change in expression is important for change of biological function whereas, for others a large expression change is required to be significant. Comparing genes at multiple levels considers these differences and identifies biologically meaningful genes that explain biological phenomena. For example, CLN3 has similar scores on PC 1, 3 and 4 in both wild-type and mutant Yeast strain. However, it has a large difference in score on PC 2, which flags it as a differentially expressed gene. None of the previously mentioned approaches identified this gene. This clearly shows that the proposed method identifies differentially expressed genes with biological basis.

## Methods

### Modeling C1 expression data using PCA

Let $X_{n\times t}^{\left(1\right)}$ be the expression data containing *n *genes measured at *t *time-points. The superscript refers to the biological condition at which the expression data is collected. Each element *x*_*ij *_represents the expression level of *i*^*th *^gene measured at the *j*^*th *^time-point. PCA decomposes the expression matrix *X*^(1)^as the sum of outer product of two vectors **z_i _**and **p_i _**plus a residual matrix **E **[30]

(1) $$X_{n\times t}^{\left(1\right)}=z_{1}^{\left(1\right)}p_{1}^{T}+z_{2}^{\left(1\right)}p_{2}^{T}+\ldots +z_{k}^{\left(1\right)}p_{k}^{T}+E$$

where $z_{i}^{\left(1\right)}$ vectors, known as scores, are of size *n *× 1, the **p_i _**vectors are called loadings and their size is *t *× 1. Here *k *≤ min(*n*, *t*).

PCA relies on the eigenvalue decomposition of the covariance matrix of *X*^(1)^, given by

(2) $$S=\frac{X^{{\left(1\right)}^{T}}X^{\left(1\right)}}{n-1}$$

provided *X*^(1)^is column mean-centered. The **p_i _**vectors are the eigenvectors of the covariance matrix of data and represent the Principal Components (directions) of variation in the data, i.e

(3) *S***p_i _**= *λ***_i_p_i_**

where *λ*_*i *_is the eigenvalue associated with the eigenvector **p_i_**. The eigenvalue *λ*_*i *_is the variance in new direction represented by **p_i_**. The Principal Components **p_i _**form an orthogonal set. Hence the score vector for each **p_i _**is given by

(4) $$z_{i}^{\left(1\right)}=X^{\left(1\right)}p_{i}$$

The Principal Components (PCs) are similar to the eigengenes of Alter et al. [19] that represent the fundamental patterns of the gene expression program that contribute to the expression of genes all over the genome. In this model (Eq 1), the expression profile of each gene is represented as a linear combination of the PCs with associated gene-specific scores. So, the expression dataset can be reconstructed if all the pairs of score and loading vectors are retained. The ($z_{i}^{\left(1\right)}$**p_i_**) pairs are arranged in descending order of *λ*_*i*_. So, the first few components associated with larger variance represent the systematic variation in data whereas components with lower variance essentially contain noise due to uncontrolled experimental and instrumental variations. The filtering of the insignificant components removes noise from the expression data and enables a meaningful comparison of the expression profiles.

The identification of significant components translates to selecting a value for *k*, the number of PCs to be retained. The simplest approach is to find the number of PCs that can capture at least a predefined amount (say 95%) of the original variance in the data. Another technique, scree test, plots the eigenvalues in non-increasing order to finds the 'knee' between dominant and insignificant PCs. The number of PCs can also be found by significance tests [31]. In this paper, we use the cross-validation procedure proposed by Wise and Ricker [32]. In this procedure, the dataset is divided into a predefined number of equal sized segments. PCA model is developed on all but one of the segments. The developed PCA model is used to reconstruct the un-modeled data. The error in reconstruction, the root-mean-square error of cross-validation (RMSECV), is plotted as function of number of PCs and the number of PCs, *k*, is selected with minimum RMSECV.

### Projection of expression data on PCA model

Through the above, a PCA model of *C*_1 _expression is generated where the expression profile of each gene over time, *x*_*i *_is represented as a combination of PCs. The expression data from condition *C*_2 _can then be compared for statistically significant differences from this PCA model. Let the expression data from *C*_2 _be denoted as $X_{n\times t}^{\left(2\right)}$ where the same genes are measured at the same time points in a different biological condition *C*_2_. If there are differences in the time points between *C*_1 _and *C*_2_, it can be addressed by resampling either/both *C*_1 _and *C*_2_. Projection of *X*^(2) ^on to the PCA model gives the corresponding scores vectors

(5) $$z_{i}^{\left(2\right)}=X^{\left(2\right)}p_{i},i\in [1,k]$$

Genes whose expression is not significantly altered in *C*_2 _will have approximately the same scores, *i*.*e*. $z_{i}^{\left(1\right)}\approx z_{i}^{2}$, while differentially expressed genes will have significant differences in their **z_i_**s. We use a statistical test to find the significance of the difference in scores and thus identify differentially expressed genes.

### Calculation of significance of differential expression

Let *Z*^Δ ^be the difference between *Z*^1 ^and *Z*^2 ^where the *i*^*th *^row of *Z*^Δ ^is the difference in the scores of gene *gi*

(6) **z_i_^Δ ^**= **z_i_^(1) ^**- **z_i_^2^**

We test the hypothesis that the differences in scores is by chance. Therefore, the null and alternative hypotheses are:

*H*_0 _= Difference in the scores of gene is by chance

*H*_1 _= Difference in scores of gene is not by chance

This hypothesis is tested based on the following insight. When we depict each gene *g*_*i *_on the scores plot, genes with small **z_i_^Δ ^**will form a *k*-dimensional cloud around the origin while genes that are differentially expressed will be away from the origin. The distance of **z_*i*_^Δ ^**from the origin measured using a suitable metric and considering the null distribution, reveals the significance of the difference in the scores, and thus that of differential expression of that gene.

The Mahalanobis distance is a common metric used with PCA and is given by

(7) $$MD_{i}^{2}=(Z_{i}^{\Delta }-\bar{Z})\Sigma^{-1}{\left(Z_{i}^{\Delta }-\bar{Z}\right)}^{T}$$

where $\bar{Z}$ is the centroid of *Z*^Δ^and Σ is the covariance matrix of *Z*^Δ^. We use the Mahalanobis distance to find the distance between each point to the centroid and use it as evidence for the differential expression. Mahalanobis distance is the most widely used distance metric with PCA analysis [30]. The larger the distance the more evidence there is to conclude that a particular gene is differentially expressed. When the difference in scores follows a multidimensional normal distribution, the Mahalanobis distance follows a *χ*^2^distribution with *k *degrees of freedom. The p-value that the differential expression occurred by chance is then given by the cumulative distribution function:

(8) $$P_{i}=1-\int_{0}^{MD^{2}}\frac{t^{(k-2)/2}e^{\left(-t)/(2\right)}}{2^{k/2}\Gamma (k/2)}$$

where Γ(*·*) is a Gamma function.

## Authors' contributions

Both SJ and RS contributed to the concept and methodology development. SJ implemented the methodology and conducted the data analysis and biological interpretation. RS supervised the study and assisted in implementation. SJ drafted the manuscript. Both authors read and approved the final manuscript.

## Supplementary Material

Additional file 1 Expression profiles of Principal Components (PCs) extracted in mouse dataset. The first two PCs model systematic changes in expression where as rest appear to have random expressions depicting noise. This indicates that modeling this dataset with 2 PCs is good. Click here for file

Additional file 2 Heatmap of the novel genes identified by the proposed method in mouse time-course dataset. Up-regulation of gene is indicated by red color and down-regulated genes are represented by green color. From this figure, it is clear that these novel genes are differently expressed between wild-type and mouse lacking HSF1 gene. Click here for file

Additional file 3 Expression profiles of Principal Components (PCs) extracted in Yeast cell-cycle dataset. PCs 1–4 have systematic changes in expression over time where as the expression profile of rest of PCs is nearly random. This indicates that modeling this dataset with 4 PCs is good. Click here for file

Additional file 4 Expression profiles of genes from CLB2 cluster that are not identified as differentially expressed by the proposed method. Solid line represents the expression profile in WT strain and the dotted line represents the expression profile in KO strain. Blue horizontal lines correspond to 2-fold change. Most (15 of 20) have less than 2-fold change in both WT and KO strains. Increasing the p-value threshold from 0.05 to 0.10 will lead to identification of 3 more genes as differentially expressed. Click here for file

Additional file 5 Expression profiles of genes from SIC1 cluster that are not identified as differentially expressed by the proposed method. Solid line represents the expression profile in the WT strain and the dotted line represents the expression profile in the KO strain. Blue horizontal lines correspond to 2-fold change. Click here for file

Additional file 6 Expression profiles of novel genes identified by EDGE method proposed by Storey et al. (2005). Solid line represents the expression profile in WT strain and the dotted line represents the expression profile in KO strain. Blue horizontal lines correspond to 2-fold change. Most of the genes have <2-fold change both in WT and KO strains and also has similar expression profiles. Click here for file

Additional file 7 Expression profiles of genes from identified as differentially expressed by Cheng et al. (2006) but not by the proposed method. Most of these genes have very little expression in both the WT and KO Yeast strains. Moreover, their expression profiles are similar in both strains. Increasing the p-value threshold from 0.05 to 0.10 will lead to identification of 6 more genes as differentially expressed by our method. Click here for file

Additional file 8 Cross-validation results for Knock-out Yeast cell-cycle. dataset. The RMSECV takes minimum value at number of PCs 5. The first 5 Principal components (PCs) captured almost 87% of the variance in the data and are used to model this dataset. Click here for file

Additional file 9 Normal distribution plots for the difference of scores on individual PCs. Normal plots of difference of scores of mouse dataset. Click here for file

Additional file 10 Normal distribution plots for the difference of scores on individual PCs. Normal plots of difference of scores of Yeast cell-cycle dataset. The coefficient of determination, *r*^2^, between the observed values and the expected values ranges from 0.92 to 0.97 indicating normal distributions for all directions. Click here for file

Additional file 11 Multivariate normal distribution plot for the difference of scores of mouse dataset. The coefficient of determination, *r*^2^, is 0.65 when all genes are used and its value increases to 0.95 after removing only 1% of outlier genes. Click here for file

Additional file 12 Multivariate normal distribution plot for the difference of scores of Yeast cell-cycle dataset. The coefficient of determination, *r*^2^, is 0.81 when all genes are used and its value increases to 0.96 after removing only 5% of outlier genes. The plots indicates that the multivariate normality assumption for the difference of scores is reasonable. Click here for file
