# Supplementary figures and images for: Principal components analysis based methodology to identify differentially expressed genes in time-course microarray data
Source: BMC Bioinformatics. 2008 Jun 6;9:267. doi: 10.1186/1471-2105-9-267 (PMC2435549; doi:10.1186/1471-2105-9-267)

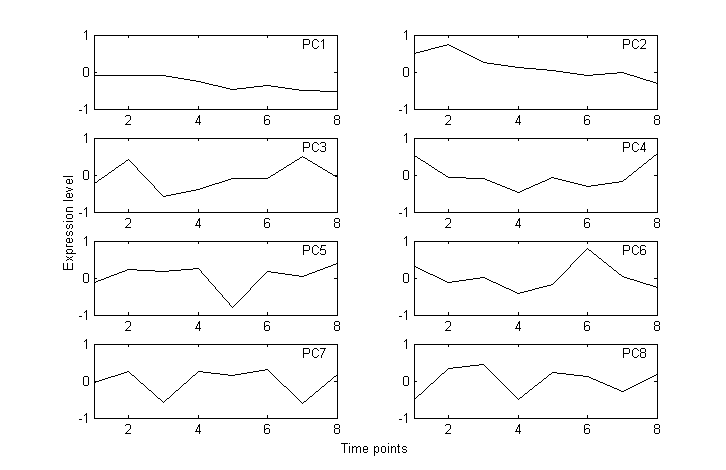

Supplement: Additional file 1 — Expression profiles of Principal Components (PCs) extracted in mouse dataset. The first two PCs model systematic changes in expression where as rest appear to have random expressions depicting noise. This indicates that modeling this dataset with 2 PCs is good. [file 1471-2105-9-267-S1.png]

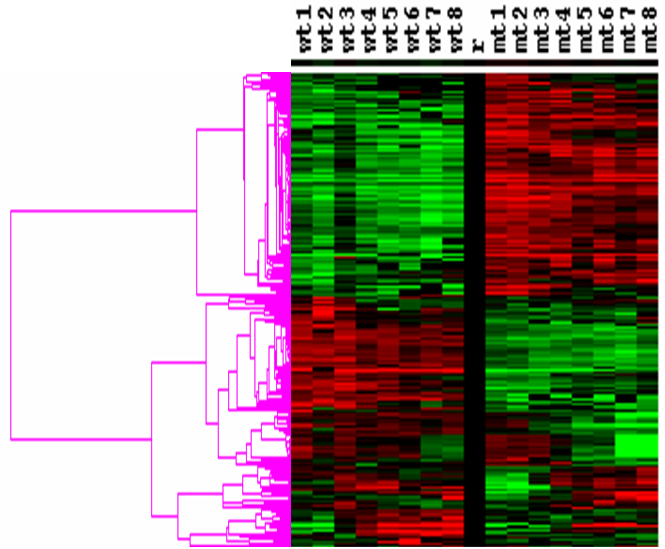

Supplement: Additional file 2 — Heatmap of the novel genes identified by the proposed method in mouse time-course dataset. Up-regulation of gene is indicated by red color and down-regulated genes are represented by green color. From this figure, it is clear that these novel genes are differently expressed between wild-type and mouse lacking HSF1 gene. [file 1471-2105-9-267-S2.png]

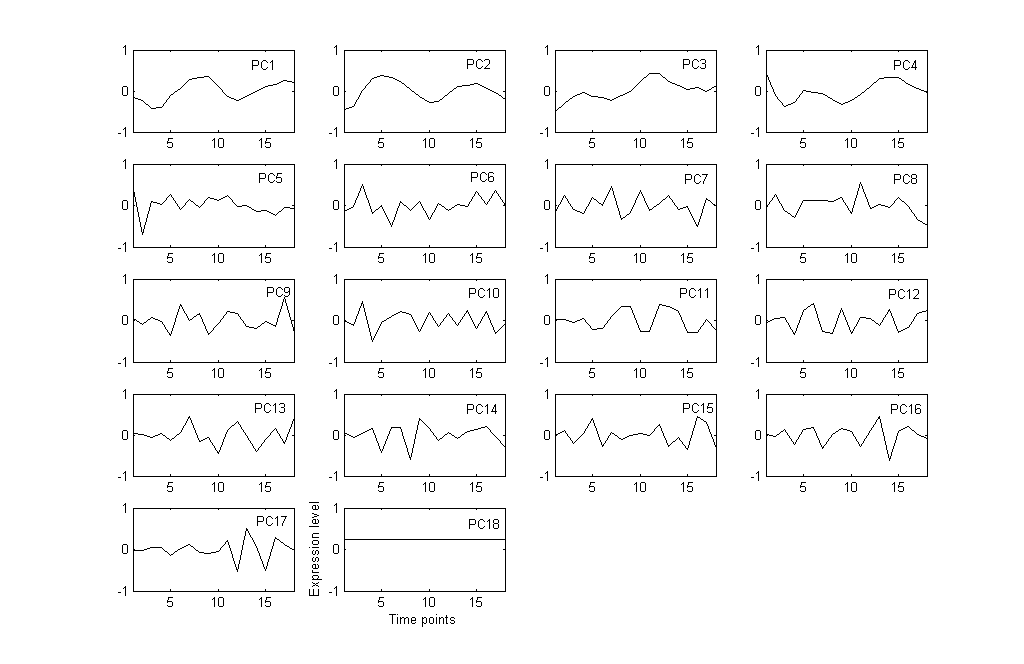

Supplement: Additional file 3 — Expression profiles of Principal Components (PCs) extracted in Yeast cell-cycle dataset. PCs 1–4 have systematic changes in expression over time where as the expression profile of rest of PCs is nearly random. This indicates that modeling this dataset with 4 PCs is good. [file 1471-2105-9-267-S3.png]

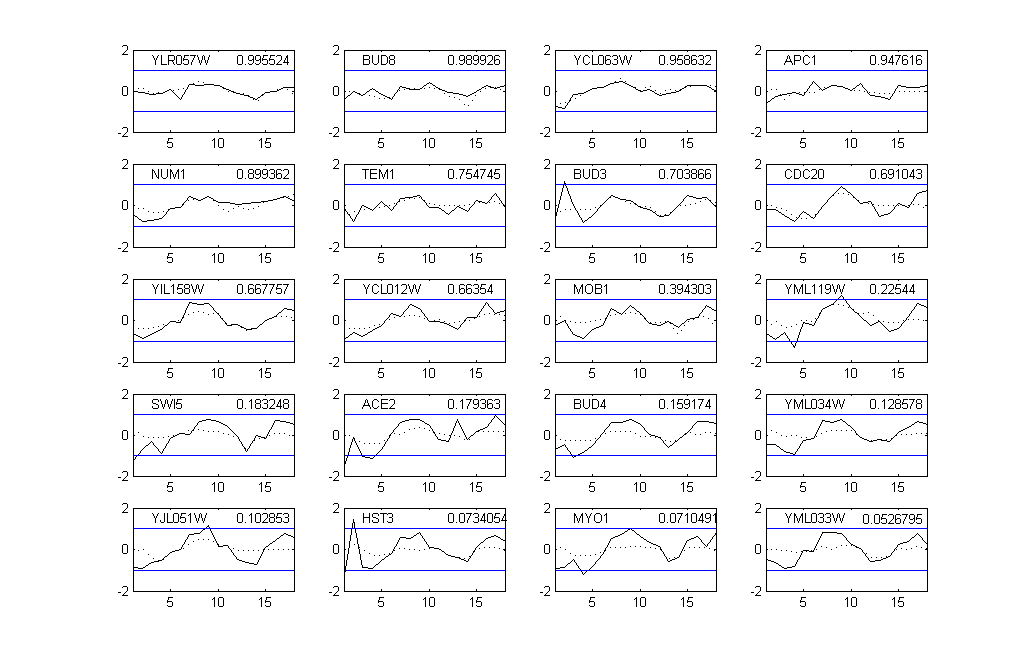

Supplement: Additional file 4 — Expression profiles of genes from CLB2 cluster that are not identified as differentially expressed by the proposed method. Solid line represents the expression profile in WT strain and the dotted line represents the expression profile in KO strain. Blue horizontal lines correspond to 2-fold change. Most (15 of 20) have less than 2-fold change in both WT and KO strains. Increasing the p-value threshold from 0.05 to 0.10 will lead to identification of 3 more genes as differentially expressed. [file 1471-2105-9-267-S4.png]

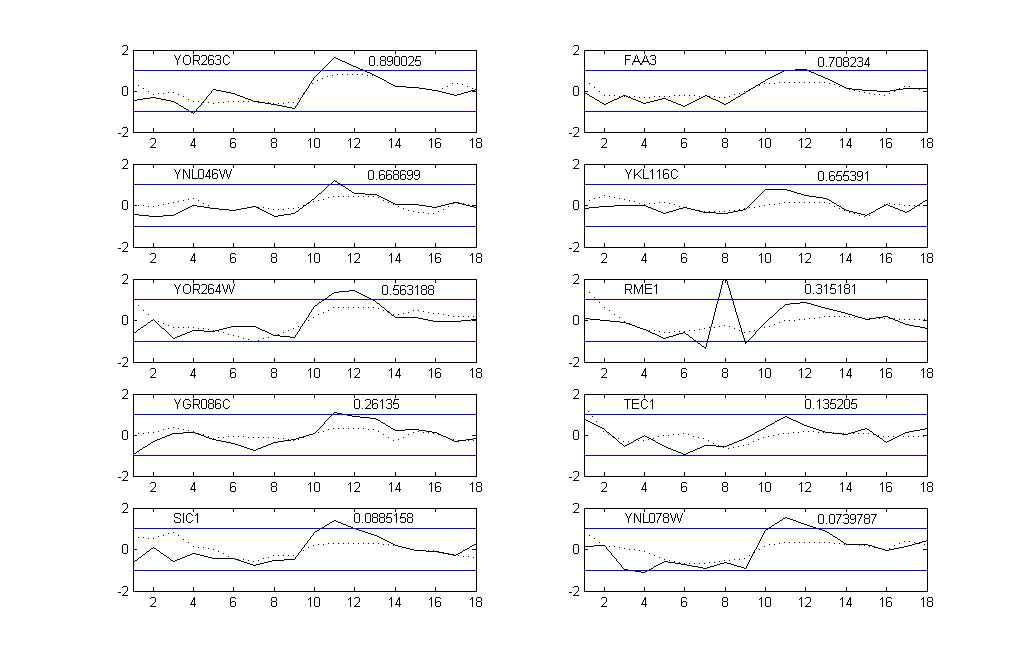

Supplement: Additional file 5 — Expression profiles of genes from SIC1 cluster that are not identified as differentially expressed by the proposed method. Solid line represents the expression profile in the WT strain and the dotted line represents the expression profile in the KO strain. Blue horizontal lines correspond to 2-fold change. [file 1471-2105-9-267-S5.png]

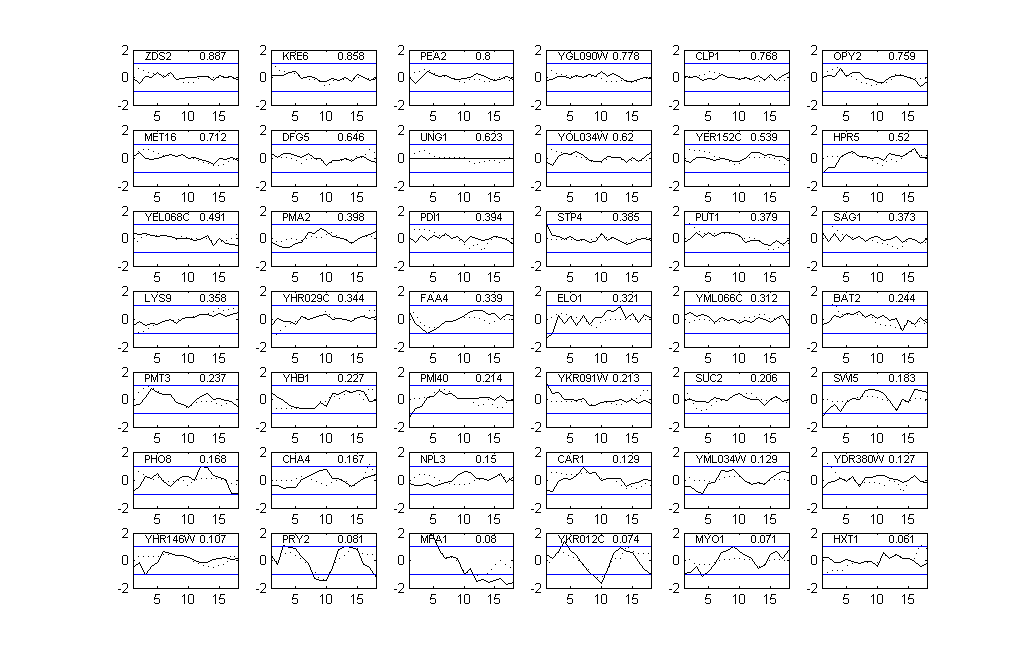

Supplement: Additional file 6 — Expression profiles of novel genes identified by EDGE method proposed by Storey et al. (2005). Solid line represents the expression profile in WT strain and the dotted line represents the expression profile in KO strain. Blue horizontal lines correspond to 2-fold change. Most of the genes have <2-fold change both in WT and KO strains and also has similar expression profiles. [file 1471-2105-9-267-S6.png]

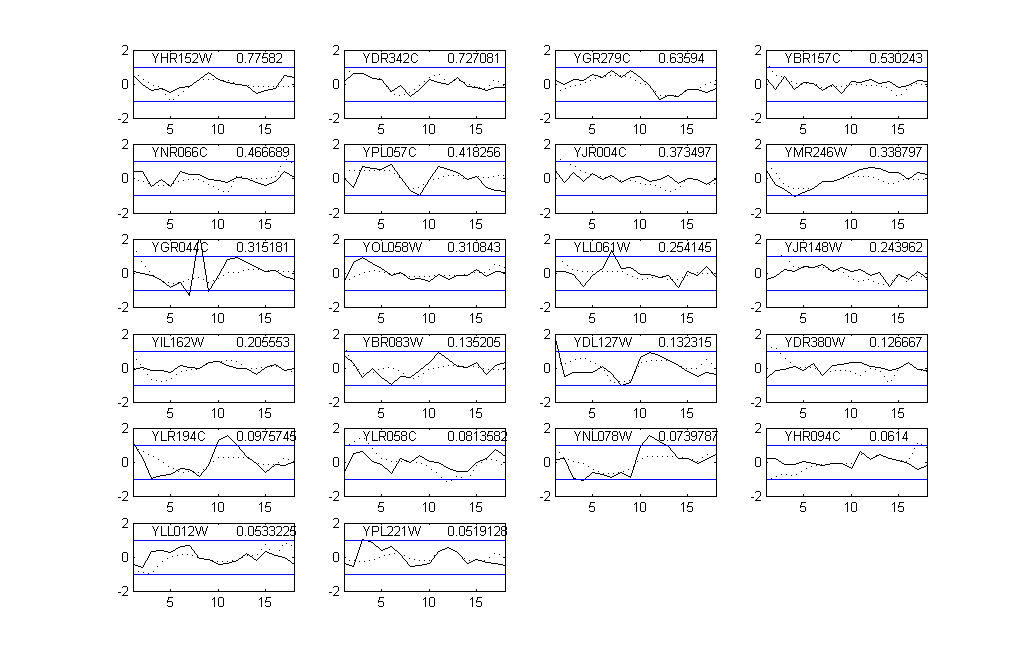

Supplement: Additional file 7 — Expression profiles of genes from identified as differentially expressed by Cheng et al. (2006) but not by the proposed method. Most of these genes have very little expression in both the WT and KO Yeast strains. Moreover, their expression profiles are similar in both strains. Increasing the p-value threshold from 0.05 to 0.10 will lead to identification of 6 more genes as differentially expressed by our method. [file 1471-2105-9-267-S7.png]

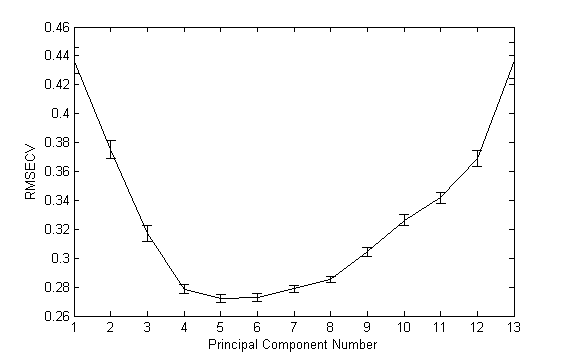

Supplement: Additional file 8 — Cross-validation results for Knock-out Yeast cell-cycle. dataset. The RMSECV takes minimum value at number of PCs 5. The first 5 Principal components (PCs) captured almost 87% of the variance in the data and are used to model this dataset. [file 1471-2105-9-267-S8.png]

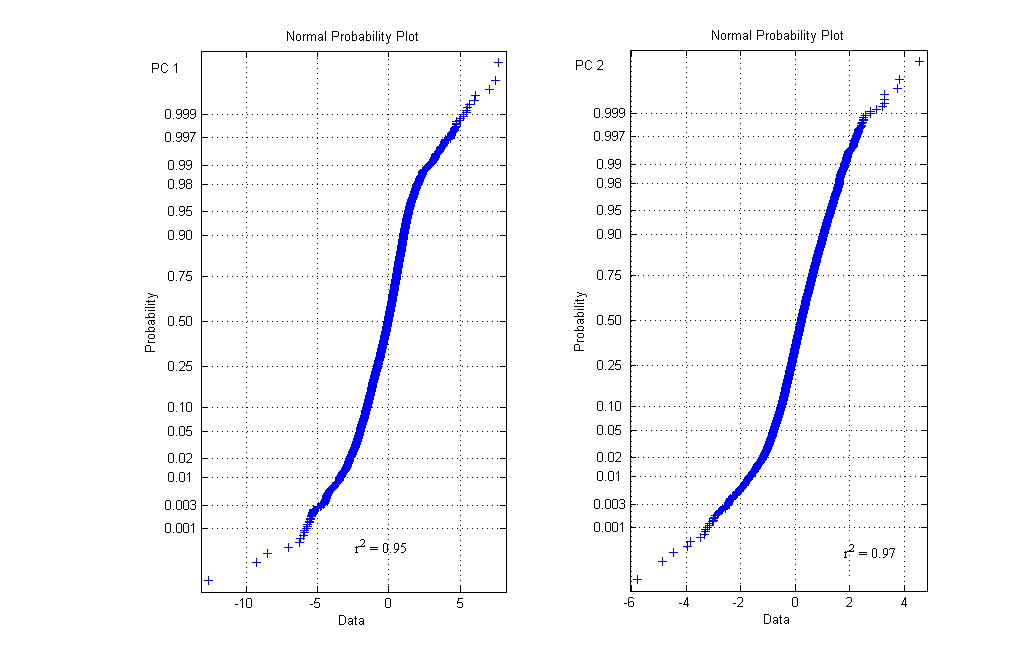

Supplement: Additional file 9 — Normal distribution plots for the difference of scores on individual PCs. Normal plots of difference of scores of mouse dataset. [file 1471-2105-9-267-S9.png]

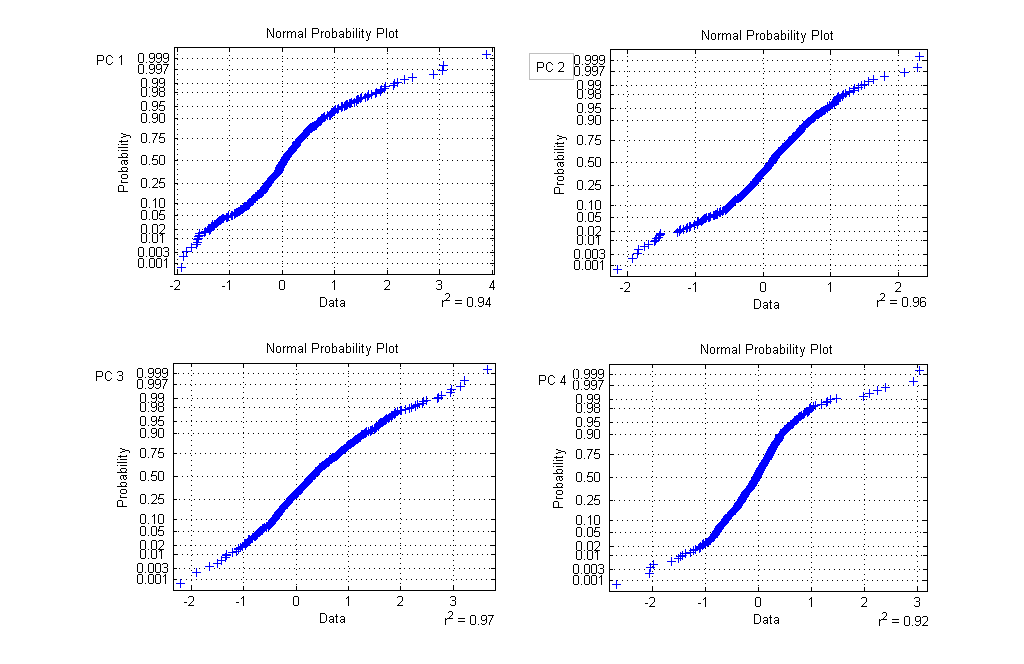

Supplement: Additional file 10 — Normal distribution plots for the difference of scores on individual PCs. Normal plots of difference of scores of Yeast cell-cycle dataset. The coefficient of determination, r2, between the observed values and the expected values ranges from 0.92 to 0.97 indicating normal distributions for all directions. [file 1471-2105-9-267-S10.png]

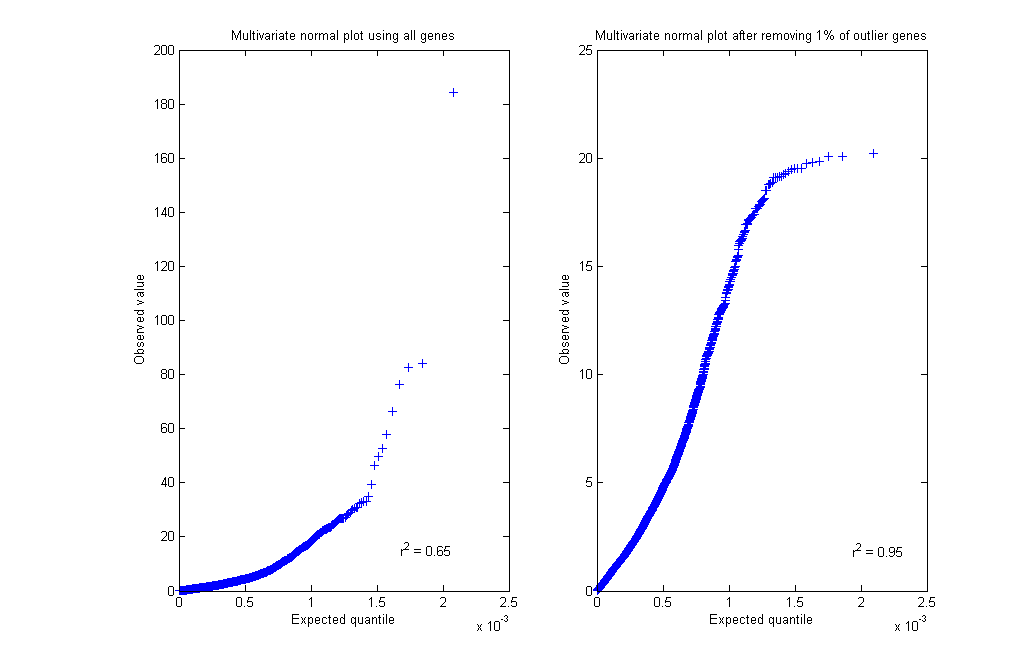

Supplement: Additional file 11 — Multivariate normal distribution plot for the difference of scores of mouse dataset. The coefficient of determination, r2, is 0.65 when all genes are used and its value increases to 0.95 after removing only 1% of outlier genes. [file 1471-2105-9-267-S11.png]

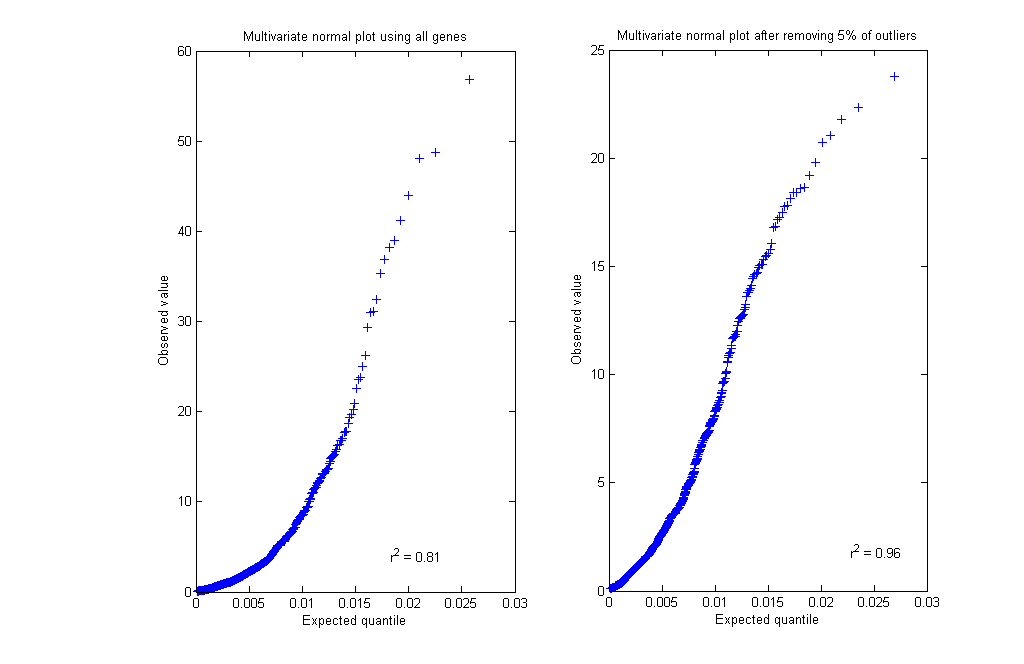

Supplement: Additional file 12 — Multivariate normal distribution plot for the difference of scores of Yeast cell-cycle dataset. The coefficient of determination, r2, is 0.81 when all genes are used and its value increases to 0.96 after removing only 5% of outlier genes. The plots indicates that the multivariate normality assumption for the difference of scores is reasonable. [file 1471-2105-9-267-S12.png]
